# Impact of COVID-19 Lockdown on Physical Activity Among the Chinese Youths: The COVID-19 Impact on Lifestyle Change Survey (COINLICS)

**DOI:** 10.3389/fpubh.2021.592795

**Published:** 2021-02-04

**Authors:** Junmin Zhou, Xiaofen Xie, Bing Guo, Rong Pei, Xiaofang Pei, Shujuan Yang, Peng Jia

**Affiliations:** ^1^West China School of Public Health and West China Fourth Hospital, Sichuan University, Chengdu, China; ^2^School of Health Caring Industry, Sichuan University of Arts and Science, Dazhou, China; ^3^International Institute of Spatial Lifecourse Epidemiology (ISLE), Hong Kong, China; ^4^Department of Land Surveying and Geo-Informatics, The Hong Kong Polytechnic University, Hong Kong, China

**Keywords:** COVID-19, physical activity, sedentary behavior, youth, China

## Abstract

**Background:** The study sought to assess the changes in physical activity (PA) and sedentary time among Chinese youths at different stages after the COVID-19 outbreak.

**Methods:** It was based on a retrospective online survey conducted in May 2020. More than 10,000 youths voluntarily recalled their PA-related information at three stages: before COVID-19 (January), during lockdown (February), and after lockdown (May). χ^2^ tests were conducted to evaluate the significance of the differences in participants' characteristics between sexes, and Wilcoxon Rank Sum tests were performed to examine the significance of differences in changes in PA and sedentary behavior levels between sexes.

**Results:** A total of 8,115 participants were included, with a mean age of 20. The percentage of no PA per week increased significantly and then slightly fell, and that of ≥150 min/week substantially decreased and then rebounded partially (all *p* < 0.001) (for instance, the percentage of ≥150 min/week of PA total decreased from 38.6 to 19.4%, then rebounded back to 25.3%). Means hours per day spent in sedentary behaviors had significantly increased during lockdown comparing to pre-COVID-19 (all *p* < 0.001). There were more participants reported reduced PA level than those indicated increased, and more participating youths had their sedentary behavior level increased than those who had it decreased.

**Conclusions:** The study found COVID-19 had both immediate and longer-term impacts on self-reported physical activities and sedentary behaviors among Chinese youths. Relevant efforts should be strengthened to get youths physically moving again.

## Introduction

The modern post-industrial life to date has featured physical inactivity and sedentary behavior, which has been a global pandemic resulting in huge costs for human beings (1). Physical inactivity and sedentary behavior have been associated with a large number of mental and physical chronic diseases, including but not limited to depression, heart diseases, stroke, diabetes, and cancers (2–4). A lot of efforts have been made to address the physical inactivity and sedentary behavior for years. However, this issue appears to persist (5, 6), as physical activity (PA) patterns did not improve and time spent on sedentary behavior has significantly increased over time (6). At the current pace, the 2025 global PA target (reducing 10% of insufficient PA) set by the World Health Organization member states would not be met (5).

However, the current situation may have been worsened by the stay-at-home order, issued by many governments around the world aiming at containing the spread of the coronavirus disease 2019 (COVID-19) pandemic, which has brought a significant impact on human lives. Millions of people are thus affected globally. Although some comments and reviews have indicated that PA will be essential for mental and physical health during the lockdown period (7–10), a few studies examining impacts of COVID-19 pandemic on PA and sedentary behavior have revealed that PA had substantially decreased while sedentary time had significantly increased during the lockdown in children and adolescents (11–13). However, it appears that such evidence is missing in youth, who is particularly vulnerable to lifestyle changes (14). Moreover, another important question we should answer is about the longer-term impact of COVID-19 pandemic on PA and sedentary behavior patterns; e.g., could the PA level, if affected by lockdown measures, return to normal after lockdown is lifted (9)? If the immediate impacts last and become new social norms, the impacts of COVID-19 on PA and sedentary behavior reduction could be catastrophic, and further aggressive efforts then need to be taken.

Therefore, the aim of this study was to assess the immediate and longer-term changes in PA and sedentary behavior in youths by using a large sample from all provinces of China. Our timely results would draw special attention from a wide array of stakeholders, from clinical practitioners to policy-makers, to the changed PA and sedentary behaviors among youths, so that these influences could be considered and hopefully remedied through clinical practice and policy interventions during this unusual period.

## Methods

### Data Collection

This study was based on the COVID-19 Impact on Lifestyle Change Survey (COINLICS), a retrospective online survey using a self-administered questionnaire distributed via social media platforms in May 2020. Recalled information was collected from three stages: before COVID-19 (January 2020), lockdown (February 2020), and 3 months after lockdown lifted (May 2020). The inclusion criteria were: (1) at post-mandatory education level (high school students, junior college students, undergraduate and postgraduate students); (2) residents of China and speak Chinese; (3) own a smartphone with internet access.

A web-based questionnaire was initially distributed among three Tencent QQ groups and three WeChat groups of educators at three education levels (high school, college, and graduate school). At least two educators from each province had shared the online questionnaire with their students through Tencent QQ and WeChat groups and/or moments. Those who completed the questionnaire were also encouraged to forward it to others. Informed consent of all participants was collected on the online questionnaire. Only those who agreed to participate and clicked the “agree” button could continue the questionnaire. Three commonsensical questions were used in the questionnaire to check the validity of the questionnaire (e.g., where is the capital of China). If any of the three questions were answered incorrectly, the questionnaire was considered as invalid. The questionnaire was required to be completed online anonymously. It usually took 10–20 min to complete the questionnaire. The study was approved by Sichuan University Medical Ethical Review Board (KS2020414).

A total of 10,082 participants were recruited from all Chinese provinces using snowball sampling from May 9 to May 24, 2020. In the study, 1,967 of them were excluded due to the exclusion criteria we applied (i.e., PA time + sedentary behavior time + sleep time ≤ 24 h/days), so the final sample size was 8,115. Since all questions were required, there was no missing data.

### Measurement

International Physical Activity Questionnaire (IPAQ)—long form was used to measure the physical activities and sedentary activities (15). Since all participants were full-time students, the occupational PA domain was removed. For each domain of PA (i.e., transportation, housework, and leisure-time), a score was summed up separately. Specifically, the number of minutes of moderate activity (including walking and riding) and two times of the number of minutes of vigorous activity were added up in each domain (16). Then, a total PA score was calculated by adding the scores of all domains. Moderate activities are defined as those performed for at least 10 min that produce a moderate increase in respiration and heart rate, and cause sweating, while vigorous activities are those producing a greater increase in respiration, heart rate, and sweating. According to the guidelines of the US Department of Health and Human Services (17), sufficient PA was defined as ≥150 min/week. The PA was categorized into three levels: none, 1–149 min/weeks, and ≥150 min/weeks (18). Changs in PA levels were grouped into: increased, constant, and decreased, while increased indicates moving from lower levels to higher levels, decreased represents moving from higher levels to lower levels, and constant means staying in the same levels. The same rule applied to changes in sedentary behavior levels.

Information regarding sedentary behavior was collected by the IPAQ, too. Average time spent in sedentary behaviors (e.g., watching TV) during the three stages was recorded for workdays and weekends separately.

In addition to the IPAQ-long form, the variables that were also collected were: sex, age, ethnicity, urbanicity, region, household income, major and current education level.

### Statistical Analysis

Descriptive statistics were calculated as mean and standard deviation (SD) for continuous variables and percentages for categorical variables. χ^2^ tests were conducted to evaluate the significance of differences in participants' characteristics between sexes, and Wilcoxon Rank Sum tests were performed to examine the significance of differences in changes in PA and sedentary behavior levels (under lockdown vs. pre-COVID-19 and lockdown lifted vs. pre-COVID-19) between sexes. In addition, Cuzick's tests for trend were carried out to determine the associations between PA and sedentary behavior levels and the different stages of COVID-19. Furthermore, to understand the association between participants' characteristics and changes in PA total levels (decreased vs. constant), multivariable logistic regressions were conducted. All statistical analyses were performed using R 3.6.2 and statistical significance was declared if *p* < 0.05 (19).

## Results

In the study, a total of 8,115 participants were included, with an average age of 20 (ranging from 15 to 33). As shown in Table 1, there were more females than males (5,688 vs. 2,427). Han people dominated our sample (over 95% were Han ethnicity). A slightly more non-urban sample than urban sample was collected. The most majority were from western China. Males were more likely to be majoring in science or engineering (50.8%), while females tended to be majoring in medical science (39.4%) and social science (43.1%) (*p* < 0.001). In terms of the current education level, undergraduate students were the majority (76.7% in males and 72.6% in females).

**Table 1:** Baseline characteristics of participating youths (*n* = 8,115).

**Variable**	**Male** ** (*n =* 2,427)**	**Female** ** (*n =* 5,688)**	**Total** ** (*n =* 8,115)**	***p*-value**
**Ethnicity** ***n*** **(%)**^**a**^				0.309
Han	2,319 (95.6)	5,403 (95.0)	7,722 (95.2)	
Minority	108 (4.4)	285 (5.0)	393 (4.8)	
**Urbanicity** ***n*** **(%)**^**a**^				0.040
Urban	1,024 (42.2)	2,261 (39.8)	3,285 (40.5)	
Non-urban	1,403 (57.8)	3,427 (60.2)	4,830 (59.5)	
**Region** ***n*** **(%)**^**a**^				0.252
Northeast	10 (0.4)	22 (0.4)	32 (0.4)	
East	220 (9.1)	559 (9.8)	779 (9.6)	
West	2,090 (86.1)	4,909 (86.3)	6,999 (86.2)	
Central	93 (3.8)	176 (3.1)	269 (3.3)	
Hubei	14 (0.6)	22 (0.4)	36 (0.4)	
**Household income (RMB/year)** ***n*** **(%)**^**a**^				<0.001
<12,000	475 (19.6)	1,040 (18.3)	1,515 (18.7)	
≥12,000–20,000	541 (22.3)	1,660 (29.2)	2,201 (27.1)	
≥20,000–60,000	643 (26.5)	1,537 (27.0)	2,180 (26.9)	
≥60,000–10,0000	386 (15.9)	755 (13.3)	1,141 (14.1)	
≥100,000–200,000	259 (10.7)	511 (9.0)	770 (9.5)	
≥200,000	123 (5.1)	185 (3.3)	308 (3.8)	
**Major** ***n*** **(%)**^**a**^				<0.001
Medical Science	555 (22.9)	2,241 (39.4)	2,796 (34.5)	
Science or Engineering	1,232 (50.8)	996 (17.5)	2,228 (27.5)	
Social Science	640 (26.4)	2,451 (43.1)	3,091 (38.1)	
**Current education** ***n*** **(%)**^**a**^				<0.001
High school	508 (20.9)	1,380 (24.3)	1,888 (23.3)	
Undergraduates	1,861 (76.7)	4,132 (72.6)	5,993 (73.9)	
Graduates	58 (2.4)	176 (3.1)	234 (2.9)	

a *Percentage within sex*.

*χ^2^ tests were conducted to evaluate the significance of differences in participants' characteristics between sexes*.

**Figure 1:**
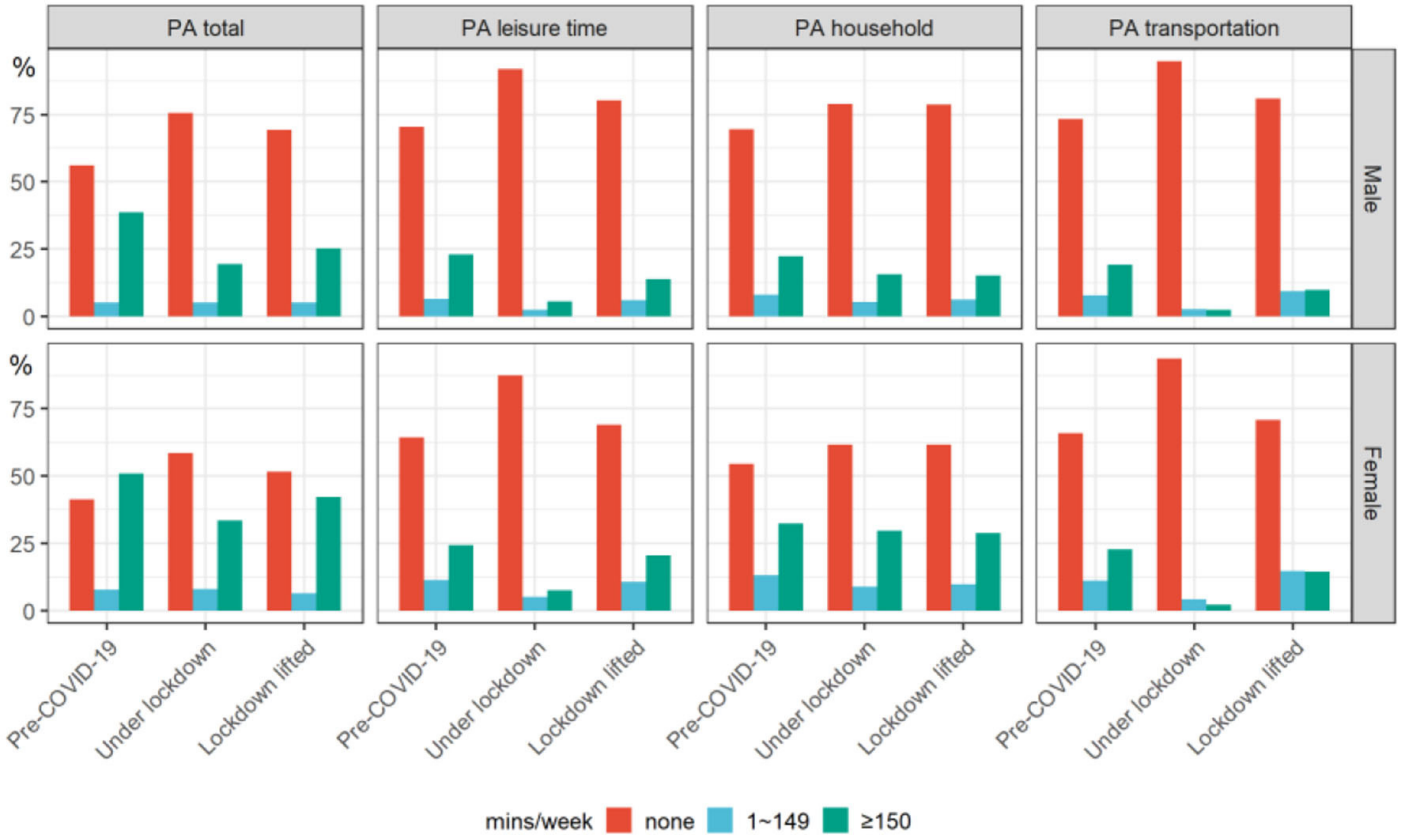
Physical activity in different stages of COVID-19 (pre-COVID-19, under lockdown, and 3 months after lockdown lifted) by sex among participating youths. Physical activity (none, 1–149 min/weeks, and ≥150 min/weeks) was significantly associated with stages of COVID-19 in four domains (PA total, PA leisure time, PA household, and PA transportation) for both male and female (*p* < 0.001). See Supplementary Table 1 for more details. PA, physical activity.

As shown in Figure 1, PA pattern was significantly associated with stages of COVID-19 in four domains (PA total, PA leisure time, PA household, and PA transportation) for both males and females. Specifically, the percentage of none PA per week increased significantly and then slightly fell, and that of ≥150 min/weeks substantially decreased and then rose partially (all *p* < 0.001, see Supplementary Table 1). For example, the percentage of reporting ≥150 min/weeks in males was 38.6% in Pre-COVID-19, then dropped almost half during lockdown (19.4%), before it recovered slightly (25.3%) after lockdown lifted (*p* < 0.001).

**Figure 2:**
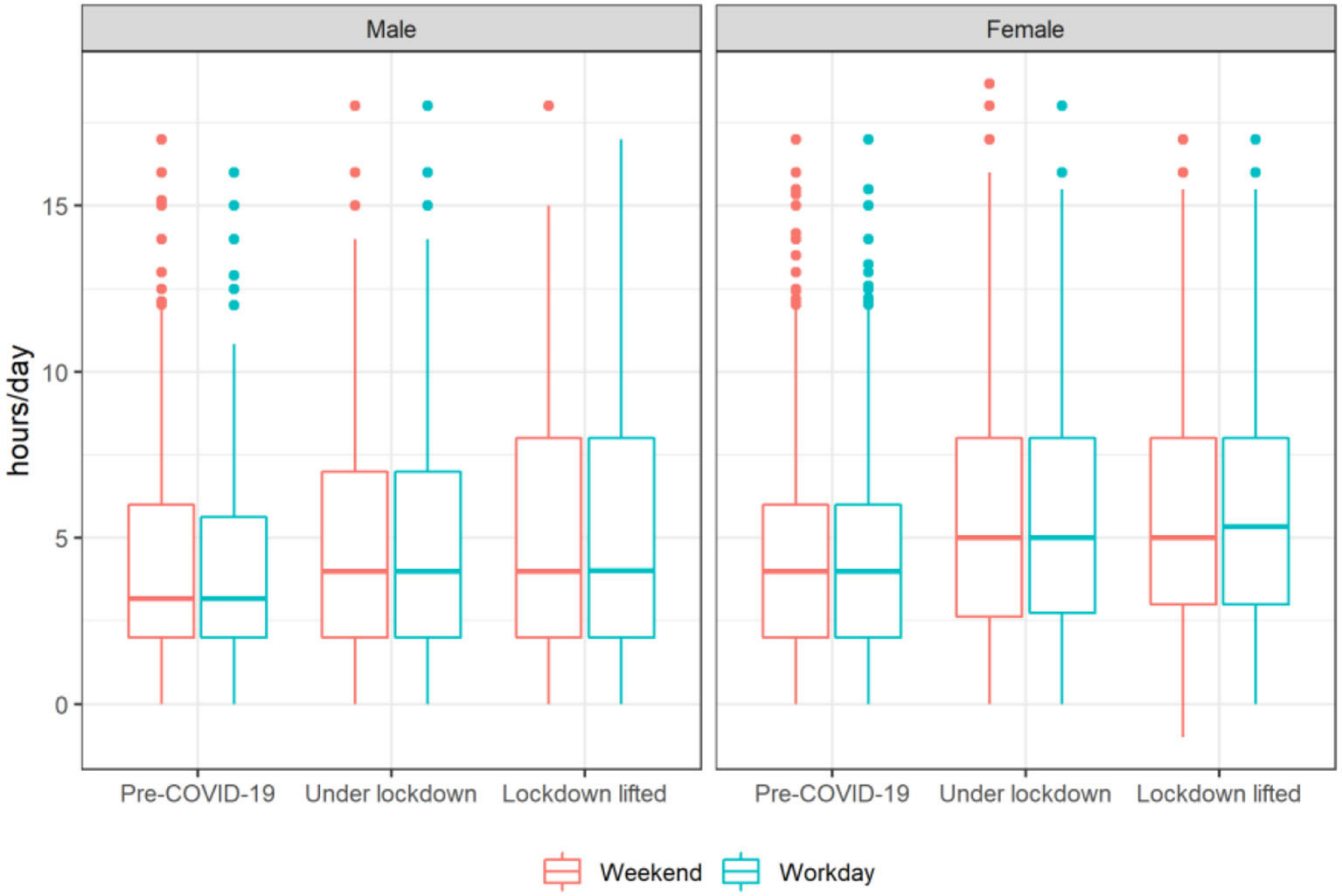
Time spent in sedentary behaviors in different stages of COVID-19 (pre-COVID-19, under lockdown, and 3 months after lockdown lifted) by sex among participating youths. Time spent in sedentary behaviors in different stages of COVID-19 was different on weekend and workday for both male and female (*p* < 0.001). See Supplementary Table 1 for more details.

Figure 2 shows time spent in sedentary behaviors before COVID-19, during lockdown, and after 3 months of lockdown lifted for both males and females. On both weekends and workdays, means of hours per day spent in sedentary behaviors had significantly increased during lockdown comparing to pre-COVID-19. Nevertheless, it did not fall back in May 2020 and even slightly increased (all *p* < 0.001, see Supplementary Table 1). For example, the average time spent in sedentary behaviors on a workday in women was 4.3 h, then increased to 5.1 h, before it finally jumped to 5.5 (*p* < 0.001).

Table 2 presents the changes in PA and sedentary behavior levels during lockdown vs. pre-COVID-19, and changes in PA and sedentary behavior after lockdown lifted vs. pre-COVID-19. While most of the participants remained constant in terms of PA level and sedentary behavior level between during lockdown and pre-COVID-19 and between lockdown lifted and pre-COVID-19, there were more participants reported reduced PA level than those indicated increased PA level (for all PA domains and PA total), and more participating youths had their sedentary behavior level increased than those who had it decreased. For instance, 24.7% of males reported decreased PA total level between during lockdown and pre-COVID-19, but the percentage of reported increased PA total level was as low as 3.5%. The changes in PA total after lockdown lifted vs. pre-COVID-19, and changes in sedentary time for weekend and workday during lockdown vs. pre-COVID-19 and after lockdown lifted vs. pre-COVID-19 were significantly different between males and females (*p* < 0.05).

**Table 2:** Changes in physical activity and sedentary behavior levels during lockdown vs. pre-COVID-19, and changes in PA and sedentary behavior after lockdown lifted vs. pre-COVID-19 among participating youths.

	**Under lockdown—Pre-COVID-19**			***p*-value**	**Lockdown lifted – Pre-COVID-19**			***p*-value**
	**Male** ** (*n =* 2427, row percentage)**	**Female** ** (*n =* 5688, row percentage)**	**Total** ** (*n =* 8115, column percentage)**		**Male** ** (*n =* 2427, row percentage)**	**Female** ** (*n =* 5688, row percentage)**	**Total** ** (*n =* 8115, column percentage)**	
**PA total %**				0.449				0.002
Increased	16.1	83.9	3.5		20.7	79.3	7.6	
Constant	30.9	69.1	71.8		30.6	69.4	72.7	
Decreased	29.1	70.9	24.7		30.8	69.2	19.7	
**PA leisure time %**				0.139				<0.001
Increased	14.2	85.8	2.6		16.9	83.1	10.2	
Constant	31.7	68.3	70.9		32.1	67.9	72.2	
Decreased	26.6	73.4	26.5		28.6	71.4	17.5	
**PA household %**				<0.001				<0.010
Increased	17.7	82.3	8.3		18.3	81.7	9.9	
Constant	31.4	68.6	77.1		32.0	68.0	73.2	
Decreased	28.8	71.2	14.7		27.5	72.5	16.9	
**PA transportation %**				<0.001				0.126
Increased	26.8	73.2	1.6		18.6	81.4	9.7	
Constant	32.2	67.8	69.3		32.3	67.7	71.2	
Decreased	24.5	75.5	29.2		26.9	73.1	19.2	
**Sedentary time weekend %**				0.036				0.012
Increased	27.8	72.2	8.5		28.0	72.0	9.5	
Constant	29.9	70.1	87.8		29.8	70.2	86.1	
Decreased	35.1	64.9	3.8		36.3	63.7	4.4	
**Sedentary time workday %**				<0.001				<0.001
Increased	26.3	73.7	8.2		26.7	73.3	9.6	
Constant	29.9	70.1	87.6		29.8	70.2	85.5	
Decreased	37.9	62.1	4.2		38.4	61.6	4.9	

*PA, physical activity*.

*The categories “increased,” “constant,” and “decreased” reflect the changes of PA levels (none, 1–149 min/weeks, and ≥150 min/weeks). For instance, if one moved from ≥150 min/weeks to 1–149 min/week, then deemed as “decreased.” The same applies to sedentary time levels (<2 h/days and ≥2 h/days)*.

*χ^2^ tests were performed to examine the significance of differences in changes in PA and sedentary behavior levels between sexes*.

Our multivariable logistic regression (see Supplementary Table 2) shows that older participants (OR, 1.06; 95% CI, 1.02–1.09), those with higher household income (e.g., OR, 1.46; 95% CI, 1.23–1.73 for ≥12,000–20,000 group), and undergraduates (OR, 1.53; 95% CI, 1.25–1.87) comparing to high school students were more likely to report decreased PA level under lockdown; non-urban participants (OR, 0.80; 95% CI, 0.72–0.90) were less likely to have decreased PA level under lockdown. After lockdown lifted, higher household income groups (OR, 1.37; 95% CI, 1.16–1.63 for ≥12,000–20,000 group; OR, 1.26; 95% CI, 1.03–1.54 for ≥60,000–10,0000 group; OR, 1.54; 95% CI, 1.24–1.93 for ≥100,000–200,000 group) were more likely to report decreased PA level.

## Discussion

China, as the first country to be impacted by COVID-19 and also first to implement and lift lockdown measures, provides valuable evidence and references to other countries. This study, by using a large sample of Chinese, revealed that COVID-19 brought significant and unfavorable impact on youths' self-reported physical activities and sedentary behaviors, and such impacts were maintained for at least 3 months. Specifically, PA had substantially decreased yet sedentary time had significantly increased during the lockdown. After lockdown lifted, PA had rebounded slightly but sedentary time remained.

Our study showed that before COVID-19 the percentage of participants who reported sufficient physical activity (≥150 min/weeks) was low (38.6% in males and 50.9% in females), and the sedentary time was ~4 h. This is in line with existing literature (20). A small number of studies have examined the impacts of COVID-19 on PA and sedentary behavior. They consistently found that the COVID-19 had resulted in substantial and negative changes to physical activities and sedentary behaviors among children and adolescents (11–13, 21) and in adults (22–25). Our findings add to the existent literature that this trend was also found in youth and that the impact brought by COVID-19 was not only immediate also lasted. Based on the current trend, we believe that it will continue to sustain.

Although PA had rebounded slightly after lifting the lockdown, it appeared that lockdown lifting did not decrease sedentary time. As the youth become more cautious of infections of COVID-19 (26), outdoor activities might not be widely adopted despite the encouragement of governments and the resumption of school. They might tend to continue to minimize unnecessary activities as long as the perceived COVID-19 risk exists. Under such circumstances, special attention should be given to indoor PA interventions. Nevertheless, a previous study estimated that if the current patterns persist and become a new social norm, more efforts would be needed to reverse this alarming trend (9). This is especially true for sedentary behavior, which is difficult to change by health interventions (27). It is evidenced by our findings, that lockdown lift only slightly increased PA time but did not have an impact on sedentary behavior time.

To our knowledge, no studies had investigated the lasting impacts of pandemics on PA and sedentary behavior. However, a previous study examined the PA and sedentary behavior among children and adolescents affected by the 2011 earthquake and tsunami in Japan and found that PA had significantly decreased even after 3 years of the earthquake (28). This may be partially in line with our findings and suggests that particular monumental events could have lasting impacts on people's behaviors. Therefore, future studies may need to corroborate this and explore ways to mitigate such impacts.

The study has several limitations. First, recall bias might be introduced. To minimize such bias, questions on PA and sedentary behavior in different stages of COVID-19 were placed next to each other for participating youths to better compare. Since the study focused more on changes in PA and sedentary behavior instead of absolute values on certain time points, their answers were likely to be valid and reliable. Second, the use of the snowball sampling method through social media platforms does not allow us to generalize the findings to the entire youth group in China, not to mention their counterparts in other countries. However, it could be the most feasible way to reach as many as possible youths under such unusual circumstances. Third, participants might disengage from the survey before they complete, but such data was not recorded. So we would not know such impacts to our findings.

Despite these limitations, this study is unique in exploring the immediate and longer-term impacts of COVID-19 on PA and sedentary behavior among Chinese youths. It is the first scientific attempt in its kind to examine such impacts, and information from this timely and large-scale survey could inform multiple stakeholders in decision-making (29). Based on results from Supplementary Table 2, special attention may be given to students majoring in Science or Engineering and those who reported higher household income, as they were more likely to indicate decreased PA level after COVID-19.

## Conclusion

The study found that COVID-19 had both immediate and longer-term impacts on physical activities and sedentary behaviors among Chinese youths. Although physical activities had rebounded back slightly after 3 months of lockdown lift, it appeared that lockdown lift did not improve the situation of time spent in sedentary behaviors. Relevant efforts should be supported and strengthened to get youths physically moving again.

## Data Availability Statement

The raw data supporting the conclusions of this article will be made available by the authors, without undue reservation.

## Ethics Statement

The studies involving human participants were reviewed and approved by Sichuan University Medical Ethical Review Board. The ethics committee waived the requirement of written informed consent for participation.

## Author Contributions

JZ: conceptualization and writing—original draft preparation. JZ and SY: methodology, investigation, and visualization. BG and XX: software. BG: validation. XX: formal analysis. RP and SY: resources. XX: Data curation. XX, BG, RP, XP, SY, and PJ: writing—review & editing. PJ: supervision and project administration. JZ: funding acquisition. All authors contributed to the article and approved the submitted version.

## Conflict of Interest

The authors declare that the research was conducted in the absence of any commercial or financial relationships that could be construed as a potential conflict of interest.
