# Supplementary material for: Impact of COVID-19 Lockdown on Physical Activity Among the Chinese Youths: The COVID-19 Impact on Lifestyle Change Survey (COINLICS)
Source: Front Public Health. 2021 Feb 4;9:592795. doi: 10.3389/fpubh.2021.592795 (PMC7890242; doi:10.3389/fpubh.2021.592795)
Supplement: Supplementary file 1 [file Table_1.docx]

Supplementary File 1

Supplementary Table S1 Physical activity and sedentary behavior in different stages of COVID-19 (pre-COVID-19, under lockdown, and three months after lockdown lifted) among participating youths (n=8,115)

|  | Male  (n=2,427) | | | *p* trend | Female  (n=5,688) | | | *p* trend |
| --- | --- | --- | --- | --- | --- | --- | --- | --- |
|  | Pre-COVID-19 | Under lockdown | Lockdown lifted |  | Pre-COVID-19 | Under lockdown | Lockdown lifted |  |
| **PA total n(%)^a^** | |  |  | <0.001 |  |  |  | <0.001 |
| ≥150 min/week | 937(38.6) | 470(19.4) | 615(25.3) |  | 2,896(50.9) | 1,902(33.4) | 2,396(42.1) |  |
| 1-149 min/week | 127(5.2) | 125(5.2) | 127(5.2) |  | 442(7.8) | 457(8.0) | 365(6.4) |  |
| None | 1,363(56.2) | 1,832(75.5) | 1,685(69.4) |  | 2,350(41.3) | 3,329(58.5) | 2,927(51.5) |  |
| **PA leisure time n(%)^a^** | |  |  | <0.001 |  |  |  | <0.001 |
| ≥150 min/week | 561(23.1) | 136(5.6) | 336(13.8) |  | 1,379(24.2) | 430(7.6) | 1,158(20.4) |  |
| 1-149 min/week | 156(6.4) | 58(2.4) | 143(5.9) |  | 650(11.4) | 292(5.1) | 602(10.6) |  |
| None | 1,710(70.5) | 2,233(92.0) | 1,948(80.3) |  | 3,659(64.3) | 4,966(87.3) | 3,928(69.1) |  |
| **PA household n(%)^a^** | |  |  | <0.001 |  |  |  | <0.001 |
| ≥150 min/week | 543(22.4) | 382(15.7) | 368(15.2) |  | 1,836(32.3) | 1,682(29.6) | 1,633(28.7) |  |
| 1-149 min/week | 196(8.1) | 130(5.4) | 148(6.1) |  | 749(13.2) | 502(8.8) | 552(9.7) |  |
| None | 1,688(69.6) | 1,915(78.9) | 1,911(78.7) |  | 3,103(54.6) | 3,504(61.6) | 3,503(61.6) |  |
| **PA transportation n(%)^a^** | |  |  | <0.001 |  |  |  | <0.001 |
| ≥150 min/week | 463(19.1) | 61(2.5) | 236(9.7) |  | 1,299(22.8) | 121(2.1) | 236(9.7) |  |
| 1-149 min/week | 186(7.7) | 65(2.7) | 226(9.3) |  | 637(11.2) | 236(4.1) | 837(14.7) |  |
| None | 1,778(73.3) | 2,301(94.8) | 1,965(81.0) |  | 3,752(66.0) | 5,331(93.7) | 4,029(70.8) |  |
| **Sedentary time weekend mean±SD** | 4.1±2.9 | 4.6±3.1 | 4.7±3.1 | <0.001 | 4.3±2.7 | 5.1±3.0 | 5.2±2.9 | <0.001 |
| **Sedentary time workday mean±SD** | 4.0±2.8 | 4.6±3.1 | 4.7±3.2 | <0.001 | 4.3±2.7 | 5.1±2.9 | 5.5±3.0 | <0.001 |

PA, physical activity.

a Percentage within sex.

Note: Cuzick's tests for trend were carried out to determine the associations between PA and sedentary behavior levels and the three stages of COVID-19.

Supplementary File 2

Supplementary Table S2 Multivariable logistic regressions on association between participants’ characteristics and changes in PA total levels (decreased vs. constant)

| **Variable** | **Under lockdown – Pre-COVID-19** | **Lockdown lifted – Pre-COVID-19** |
| --- | --- | --- |
|  | **OR (95% CI)** | **OR (95% CI)** |
| **Age** | **1.06 (1.02-1.09)** | 1.02 (0.99-1.06) |
| **Ethnicity** | |  |
| Han (Ref) | 1 | 1 |
| Minority | 0.91 (0.71-1.17) | 0.89 (0.68-1.17) |
| **Urbanicity** | |  |
| Urban (Ref) | 1 | 1 |
| Non-urban | **0.80 (0.72-0.90)** | 0.92 (0.82-1.04) |
| **Household income**  (RMB/year) | |  |
| <12,000 (Ref) | 1 | 1 |
| ≥12,000-20,000 | **1.46 (1.23-1.73)** | **1.37 (1.16-1.63)** |
| ≥20,000-60,000 | **1.42 (1.20-1.67)** | 1.11 (0.93-1.33) |
| ≥60,000-10,0000 | **1.68 (1.39-2.03)** | **1.26 (1.03-1.54)** |
| ≥100,000-200,000 | **1.91 (1.54-2.35)** | **1.54 (1.24-1.93)** |
| ≥200,000 | **1.45 (1.08-1.94)** | 1.29 (0.94-1.76) |
| **Major** | |  |
| Medical Science(Ref) | 1 | 1 |
| Science or Engineering | 0.88 (0.75-1.02) | **1.19 (1.01-1.40)** |
| Social Science | 0.94 (0.81-1.09) | 1.17 (0.99-1.38) |
| **Current education** | | |
| High school (Ref) | 1 | 1 |
| Undergraduates | **1.53 (1.25-1.87)** | 1.13 (0.91-1.39) |
| Graduates | 1.29 (0.86-1.92) | 0.97 (0.62-1.50) |

Abbreviations: OR, odds ratio; CI, confidence interval

Bold values represent statistical significance

Supplementary File 3

**The questionnaire**

1. Date of birth: year month

2. Gender：

□Male

□Female

3. Height： cm；

4. Weight:

| **A.** **Weight pre-COVID-19 (one month before January 23)** | **B. Weight changes during lockdown (January 24-february 28) compared with pre-COVID-19** | **C. Weight changes after lockdown lifted (the most recent month) compared with pre-COVID-19** |
| --- | --- | --- |
| Kg | □Increased: Kg  □Constant  □Decreased: Kg | □Increased: Kg  □Constant  □Decreased: Kg |

5. Ethnic：

□Han

□Minority

6. Where do you live?

| **A. Pre-COVID-19 (one month before January 23)** | **B. Lockdown (January 24-February 28)** | **C. Lockdown lifted (the most recent month)** |
| --- | --- | --- |
| □Urban (including at schools)  □Non-urban | □Urban (including at schools)  □Non-urban | □Urban (including at schools)  □Non-urban |

8. What is the education level you are currently in?

□Undergraduate; major: □Medicine

□Science and Engineering

□Social science

□Graduate or above; major: □Medicine

□Science and Engineering

□Social science

9. How many members of your family (including yourself) living together?

__ people

10. What is the total household income of your family in the past year? (in RMB)

□<12,000

□ 12,000-19,999

□ 20,000-59,999

□ 60,000-99,999

□ 100,000-199,999

□≥200,000

**Physical activity**

**PART 1: TRANSPORTATION PHYSICAL ACTIVITY**

| These questions are about how you traveled from place to place, including to places like work, stores, movies, and so on. | | |
| --- | --- | --- |
| **A. Pre-COVID-19 (one month before January 23)** | **B. Lockdown (January 24-February 28)** | **C. Lockdown lifted (the most recent month)** |
| 11. How many days per week did you travel in a motor vehicle like a train, bus, car, or tram? | | |
| □days/week  □No travelling in a motor vehicle••••Skip to question 13A | □days/week  □No travelling in a motor vehicle•••• Skip to question 13B | □days/week  □No travelling in a motor vehicle •••• Skip to question 13C |
| 12. How much time did you usually spend on one of those days traveling in a train, bus, car, tram, or other kind of motor vehicle? | | |
| □□hours□□minutes/day | □□hours□□minutes/day | □□hours□□minutes/day |
| 13. How many days per week did you bicycle for at least 10 minutes at a time to go from place to place? | | |
| □days/week  □No bicycling from place to place••••Skip to question 15A | □days/week  □No bicycling from place to place ••••Skip to question 15B | □days/week  □No bicycling from place to place ••••Skip to question 15C |
| 14. How much time did you usually spend on one of those days to bicycle from place to place? | | |
| □□hours□□minutes/day | □□hours□□minutes/day | □□hours□□minutes/day |
| 15. How many days per week did you walk for at least 10 minutes at a time to go from place to place? | | |
| □days/week  □No walking from place to place••••Skip to PART 2 | □days/week  □No walking from place to place ••••Skip to PART 2 | □days/week  □No walking from place to place ••••Skip to PART 2 |
| 16. How much time did you usually spend on one of those days walking from place to place? | | |
| □□hours□□minutes/day | □□hours□□minutes/day | □□hours□□minutes/day |

**PART 2: HOUSEWORK, HOUSE MAINTENANCE, AND CARING FOR FAMILY**

| This section is about some of the physical activities you might have done in and around your home, like housework, gardening, yard work, general maintenance work, and caring for your family. | | |
| --- | --- | --- |
| **A. Pre-COVID-19 (one month before January 23)** | **B. Lockdown (January 24-February 28)** | **C. Lockdown lifted (the most recent month)** |
| 17. Think about only those physical activities that you did for at least 10 minutes at a time. How many days per week did you do vigorous physical activities like heavy lifting, chopping wood, shoveling snow, or digging in the garden or yard? | | |
| □days/week  □No vigorous activity in garden or yard••••Skip to question 19A | □days/week  □No vigorous activity in garden or yard••••Skip to question 19B | □days/week  □No vigorous activity in garden or yard••••Skip to question 19C |
| 18. How much time did you usually spend on one of those days doing vigorous physical activities in the garden or yard? | | |
| □□hours□□minutes/day | □□hours□□minutes/day | □□hours□□minutes/day |
| 19. Again, think about only those physical activities that you did for at least 10 minutes at a time. How many days per week did you do moderate activities like carrying light loads, sweeping, washing windows, cooking, washing clothes, and raking in the garden or yard? | | |
| □days/week  □No moderate activity in garden or yard •••• Skip to PART 3 | □days/week  □No moderate activity in garden or yard ••••Skip to PART 3 | □days/week  □No moderate activity in garden or yard ••••Skip to PART 3 |
| 20. How much time did you usually spend on one of those days doing moderate physical activities in the garden or yard? | | |
| □□hours□□minutes/day | □□hours□□minutes/day | □□hours□□minutes/day |

**PART 3: RECREATION, SPORT, AND LEISURE-TIME PHYSICAL ACTIVITY**

| This section is about all the physical activities that you did solely for recreation, sport, exercise or leisure. Please do not include any activities you have already mentioned. | | |
| --- | --- | --- |
| **A. Pre-COVID-19 (one month before January 23)** | **B. Lockdown (January 24-February 28)** | **C. Lockdown lifted (the most recent month)** |
| 21. Not counting any walking you have already mentioned, how many days per week did you walk for at least 10 minutes at a time in your leisure time? | | |
| □days/week  □No walking in leisure time••••Skip to question 23A | □days/week  □No walking in leisure time ••••Skip to question 23B | □days/week  □No walking in leisure time ••••Skip to question 23C |
| 22. How much time did you usually spend on one of those days walking in your leisure time? | | |
| □□hours□□minutes/day | □□hours□□minutes/day | □□hours□□minutes/day |
| 23. Think about only those physical activities that you did for at least 10 minutes at a time. How many days per week did you do vigorous physical activities like aerobics, running, fast bicycling, or fast swimming in your leisure time? | | |
| □days/week  □No vigorous activity in leisure time ••••Skip to question 25A | □days/week  □No vigorous activity in leisure time ••••Skip to question 25B | □days/week  □No vigorous activity in leisure time ••••Skip to question 25C |
| 24. How much time did you usually spend on one of those days doing vigorous physical activities in your leisure time? | | |
| □□hours□□minutes/day | □□hours□□minutes/day | □□hours□□minutes/day |
| 25. Again, think about only those physical activities that you did for at least 10 minutes at a time. How many days per week did you do moderate physical activities like bicycling at a regular pace, swimming at a regular pace, and doubles tennis in your leisure time? | | |
| □days/week  □No moderate activity in leisure time •••• Skip to PART 4 | □days/week  □No moderate activity in leisure time •••• Skip to PART 4 | □days/week  □No moderate activity in leisure time •••• Skip to PART 4 |
| 26. How much time did you usually spend on one of those days doing moderate physical activities in your leisure time? | | |
| □□hours□□minutes/day | □□hours□□minutes/day | □□hours□□minutes/day |

**PART 4: SEDENTARY TIME**

| These questions are about the time you spend sitting while at work, at home, while doing course work and during leisure time. This may include time spent sitting at a desk, visiting friends, reading or sitting or lying down to watch television. Do not include any time spent sitting in a motor vehicle that you have already told me about. | | |
| --- | --- | --- |
| **A. Pre-COVID-19 (one month before January 23)** | **B. Lockdown (January 24-February 28)** | **C. Lockdown lifted (the most recent month)** |
| 27. How much time did you usually spend sitting on a weekday? | | |
| □□hours□□minutes/day | □□hours□□minutes/day | □□hours□□minutes/day |
| 28. How much time did you usually spend sitting on a weekend day? | | |
| □□hours□□minutes/day | □□hours□□minutes/day | □□hours□□minutes/day |

**PART 5: SLEEPING TIME**

| These questions are about your time for sleeping, including napping. | | |
| --- | --- | --- |
| **A. Pre-COVID-19 (one month before January 23)** | **B. Lockdown (January 24-February 28)** | **C. Lockdown lifted (the most recent month)** |
| 29. How much time did you usually sleep on a weekday (including naps)? | | |
| □□hours/day | □□hours/day | □□hours/day |
| 30. How much time did you usually sleep on a weekend day (including naps)? | | |
| □□hours/day | □□hours/day | □□hours/day |
